# Inhibition of Acetylcholinesterase Modulates NMDA Receptor Antagonist Mediated Alterations in the Developing Brain

**DOI:** 10.3390/ijms15033784

**Published:** 2014-03-03

**Authors:** Ivo Bendix, Meray Serdar, Josephine Herz, Clarissa von Haefen, Fatme Nasser, Benjamin Rohrer, Stefanie Endesfelder, Ursula Felderhoff-Mueser, Claudia D. Spies, Marco Sifringer

**Affiliations:** 1 Department of Pediatrics I, Neonatology, University Hospital Essen, Essen 45122, Germany; E-Mails: meray.serdar@uk-essen.de (M.S.); josephine.herz@uk-essen.de (J.H.); ursula.felderhoff@uk-essen.de (U.F.-M.); 2 Department of Anesthesiology and Intensive Care Medicine, Charité-Universitätsmedizin Berlin, Campus Virchow-Klinikum, Berlin 13353, Germany; E-Mails: clarissa.von-haefen@charite.de (C.H.); fatme21@hotmail.de (F.N.); benjamin.rohrer@charite.de (B.R.); claudia.spies@charite.de (C.D.S.); marco.sifringer@charite.de (M.S.); 3 Department of Neonatology, Charité-Universitätsmedizin Berlin, Campus Virchow-Klinikum, Berlin 13353, Germany; E-Mail: stefanie.endesfelder@charite.de

**Keywords:** developing brain, NMDA receptor, MK801, acetylcholinesterase, extracellular matrix, BDNF, neuroprotection

## Abstract

Exposure to *N*-methyl-d-aspartate (NMDA) receptor antagonists has been demonstrated to induce neurodegeneration in newborn rats. However, in clinical practice the use of NMDA receptor antagonists as anesthetics and sedatives cannot always be avoided. The present study investigated the effect of the indirect cholinergic agonist physostigmine on neurotrophin expression and the extracellular matrix during NMDA receptor antagonist induced injury to the immature rat brain. The aim was to investigate matrix metalloproteinase (MMP)-2 activity, as well as expression of tissue inhibitor of metalloproteinase (TIMP)-2 and brain-derived neurotrophic factor (BDNF) after co-administration of the non-competitive NMDA receptor antagonist MK801 (dizocilpine) and the acetylcholinesterase (AChE) inhibitor physostigmine. The AChE inhibitor physostigmine ameliorated the MK801-induced reduction of BDNF mRNA and protein levels, reduced MK801-triggered MMP-2 activity and prevented decreased *TIMP-2* mRNA expression. Our results indicate that AChE inhibition may prevent newborn rats from MK801-mediated brain damage by enhancing neurotrophin-associated signaling pathways and by modulating the extracellular matrix.

## 1. Introduction

The effects of pharmacological agents, which are frequently used as sedatives and anesthetics in neonatal intensive care units, are partially based on their ability to reduce neuronal excitability by blocking *N*-methyl-d-aspartate (NMDA) receptor neurotransmission [1–3]. The “brain growth spurt” period is characterized by comprehensive neurogenesis and synaptogenesis during the third trimester of pregnancy up to a two-year postnatal period in the human brain [4,5]. Neonatal treatment with the non-competitive NMDA receptor antagonist MK801 (dizocilpine) is associated with activation of apoptotic cell death, inflammation, oxidative stress, dysregulation of neuronal circuit formation, impairment of cell proliferation, inhibition of neurogenesis and down-regulation of neurotrophins [6–11]. Recent studies have showed that ketamine a commonly used intravenous anesthetic in clinical anesthesia, which exerts its function as a non-competitive NMDA receptor antagonist can affect neuronal growth and development and even induce apoptosis [12–17]. Besides its enzymatic activity to hydrolyze acetylcholine [18], the protein acetylcholinesterase (AChE; EC 3.1.1.7) has non-cholinergic functions as it has been shown to be crucial for cell adhesion, neurite formation, axonal outgrowth, neuronal migration and synaptogenesis [19–23]. Cholinesterase inhibitors are able to ameliorate impaired working memory in adult rats and behavioural deficits in adult mice induced by MK801 (dizocilpine) [24,25]. We have previously demonstrated a neuroprotective effect of AChE inhibition in a neonatal rat model of oxygen toxicity [26]. These findings led to the question of whether administration of the prototypical AChE inhibitor, physostigmine, might regulate mechanisms of NMDA receptor antagonist-induced neurodegeneration in the developing brain.

Recent studies have shown that AChE inhibitors significantly increase levels of brain-derived neurotrophic factor (BDNF) in serum of Alzheimer’s disease patients and in mice after transient cerebral ischemia and reperfusion [27,28]. BDNF plays an essential role in neuronal survival, neurogenesis, synaptic plasticity, learning and memory [29–32]. In humans, BDNF levels were found to be highest in neonates with a specific BDNF increase in serum levels from birth to day four, possibly reflecting neuroprotection against perinatal stress and hypoxia [33,34].

Matrix metalloproteinases (MMPs) are involved in brain damage but also repair mechanisms in different diseases of the central nervous system (CNS) such as stroke, traumatic brain injury, Alzheimer’s disease, and multiple sclerosis via modulation of the blood-brain barrier permeability, resulting in neurovascular dysfunction and vasogenic edema [35–43]. MMPs regulate tissue inflammation in response to oxidative stress [44,45] and MMP-2 has also been shown to play a role in neuronal damage [46,47]. However, MMPs and other proteases may also play beneficial roles in the delayed phases after injury by modulating the extracellular matrix (ECM) and trophic factors in the brain and at the neurovascular interface [45,48]. The biological activity of MMPs is regulated by transcription, activation of proenzymes, and physiological inhibition by tissue inhibitors of metalloproteinases (TIMPs), a family of multifunctional secreted proteins that regulate the cell cycle in various cell types [43,49,50].

In the present study, neonatal rats were co-treated with the AChE inhibitor physostigmine, in combination with the non-competitive NMDA receptor antagonist, MK801. Molecular effects of neonatal NMDA receptor blockade in the developing rat brain with a focus on neurotrophin BDNF expression and modulation of the extracellular matrix were investigated.

## 2. Results and Discussion

### 2.1. Results

#### 2.1.1. Physostigmine Normalizes *N*-Methyl-d-aspartate (NMDA) Receptor Antagonist-Induced Increase of Acetylcholinesterase Activity

**Figure 1.**
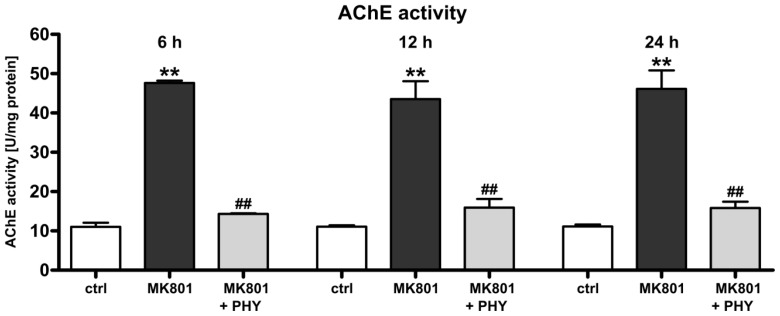
Normalization of acetylcholinesterase (AChE) activity in MK801 (dizocilpine) treated rat pups after physostigmine treatment. AChE activity, determined by an enzymatic activity assay revealed a significant increase in AChE activity at 6, 12 and 24 h after MK801 treatment (dark grey bars) whereas a single co-application of physostigmine significantly reduced AChE activity (light grey bars). Bars represent mean + SD, *n* = 6 per group, ** *p* < 0.01 compared to vehicle treated pups, ^##^ *p* < 0.01 compared to MK801 treated pups, respectively. ctrl: control; PHY: physostigmine.

As acetylcholinesterase is rapidly modulated under a variety of stress conditions and is induced during cell death [26,51–54], we investigated the regulation of AChE activity after administration of the non-competitive NMDA receptor antagonist MK801 (0.5 mg/kg at 0, 8, and 16 h) and the AChE inhibitor physostigmine (100 μg/kg at 0 h) to the immature rat brain. A marked up-regulation of AChE activity in brain hemispheres of rat pups at 6 (47.63 + 0.24 U/mg protein), 12 (43.50 + 1.87 U/mg protein) and 24 h (46.13 + 1.92 U/mg protein) after the last MK801 treatment (dark grey bars) was detected (Figure 1). In the brains of the co-treated animals, enzymatic activity was normalized to levels of vehicle treated control animals (white bars) after a single physostigmine administration (light grey bars).

#### 2.1.2. Acetylcholinesterase (AChE) Inhibition Increases Brain-Derived Neurotrophic Factor (BDNF) Expression

**Figure 2.**
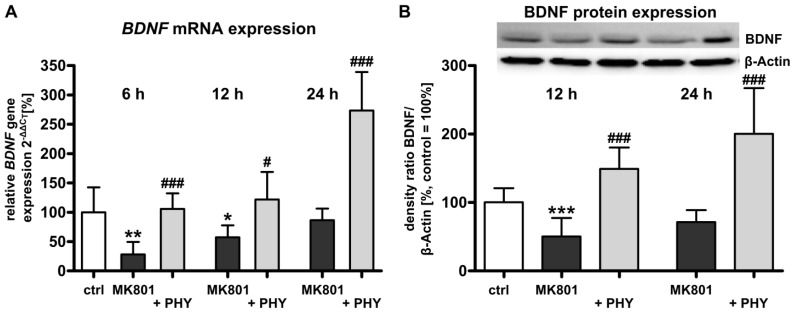
Increased brain-derived neurotrophic factor (BDNF) expression after physostigmine co-treatment in MK801 treated rat pups. (**A**) Quantitative analysis of brain *BDNF* mRNA expression at 6, 12 and 24 h after MK801 treatment (dark grey bars) with or without systemic physostigmine co-application (light grey bars); and (**B**) Quantitative protein expression of BDNF at 12 and 24 h after MK801 treatment (dark grey bars) in combination with AChE inhibition (light grey bars). The densitometric data represent the ratio of the pixel intensities of BDNF signals to the corresponding β-actin signals. Data are normalized to levels of vehicle treated pups ((control; white bar, 100%); bars represent mean + SD, *n* = 6–7 per group, *** *p* < 0.001, ** *p* < 0.01, * *p* < 0.05 compared to vehicle treated pups, ^###^ *p* < 0.001, ^#^ *p* < 0.05 compared to MK801 treated pups, respectively). ctrl: control; PHY: physostigmine.

As neonatal MK801 exposure reduces mRNA and protein expression levels of the neurotrophin BDNF [6,8], we investigated whether the AChE inhibitor physostigmine might regulate BDNF expression after pharmacological NMDA receptor blockade in the developing rat brain. Quantitative analysis of mRNA expression by real-time polymerase chain reaction (PCR) (Figure 2A) revealed a significant down-regulation of *BDNF* mRNA expression in brain hemispheres of rat pups at 6 (28.3% + 8.7%) and 12 h (57.2% + 8.4%) after MK801 treatment (dark grey bars). A single physostigmine co-administration triggered a significant increase of *BDNF* expression (light grey bars, 6 h: 105.7% + 10.8%, 12 h: 121.9% + 19.2%, and 24 h: 273.3% + 26.9%). Analysis of BDNF protein expression by Western blot was in accordance with real-time PCR results, *i.e.*, protein expression of BDNF was considerably reduced at 12 h (50.4% + 11.0%) and a single co-injection of physostigmine up-regulated BDNF protein expression (149.3% + 12.8%, Figure 2B).

#### 2.1.3. Physostigmine Modulates MK801 (Dizocilpine)-Induced Increase of Matrix Metalloproteinase (MMP)-2 Activity

**Figure 3.**
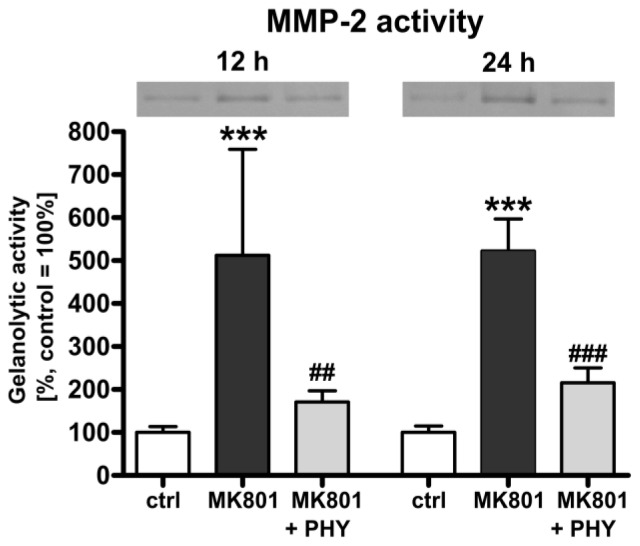
Increased matrix metalloproteinase (MMP)-2 activity after MK801 treatment is ameliorated through AChE inhibition. Gelanolytic MMP-2 activity, determined by gelatin zymography revealed a significant increase in MMP-2 activity at 12 and 24 h after MK801 treatment (dark grey bars) whereas a single co-administration of physostigmine significantly reduced MMP-2 activity (light grey bars). Data are normalized to levels of vehicle treated pups ((control; white bars, 100%); bars represent mean + SD, *n* = 6 per group, *** *p* < 0.001 compared to vehicle treated pups, ^###^ *p* < 0.001, ^##^ *p* < 0.01 compared to MK801 treated pups, respectively). ctrl: control; PHY: physostigmine.

In order to confirm the role of MMP-2 which releases pro-BDNF from cells and converts pro-BDNF to mature BDNF [55] as a key protein mediating neuroprotection in brain damage [46,47], we further analysed the effect of MK801 treatment and AChE inhibition on MMP-2 activity in the immature rat brain. Measurement of gelanolytic MMP-2 activity (Figure 3) showed a significant up-regulation of MMP-2 activity in brain hemispheres of rat pups at 12 (512.2% + 100.9%) and 24 h (522.5% + 30.4%) after NMDA receptor blockade (dark grey bars). A single physostigmine co-application strongly counter-regulated this effect (light grey bars, 12 h: 171.2% + 10.7%, 24 h: 215.5% + 14.1%).

#### 2.1.4. NMDA Receptor Antagonist Mediated Reduction of *Tissue Inhibitor of Metalloproteinase-2 (TIMP-2)* Expression Is Increased by Physostigmine Co-Treatment

**Figure 4.**
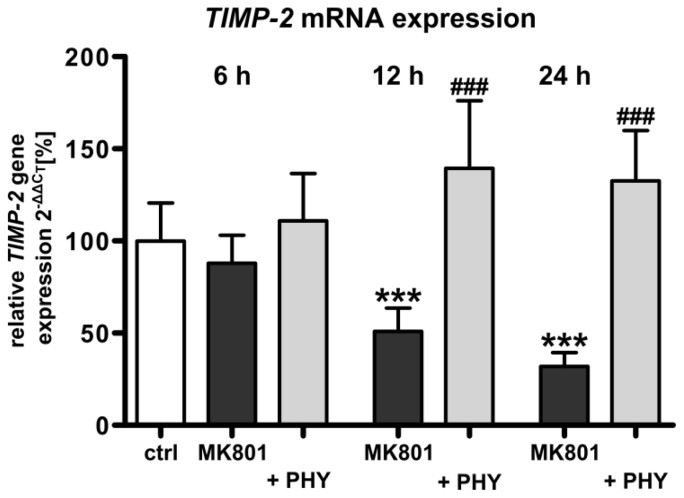
Stabilization of *TIMP-2* mRNA expression after AChE inhibition in MK801 treated rat pups. Quantitative analysis of brain *TIMP-2* mRNA expression after MK801 treatment (dark grey bars) with or without physostigmine co-application (light grey bars). Data are normalized to levels of vehicle treated pups ((control; white bar, 100%); bars represent mean + SD, *n* = 6 per group, *** *p* < 0.001 compared to vehicle treated pups, ^###^ *p* < 0.001 compared to MK801 treated pups, respectively). ctrl: control; PHY: physostigmine.

The enzymatic activity of MMPs is inactivated by the endogenous inhibitors TIMPs. As TIMP-2 is reported to be a physiologic inhibitor of MMP-2 [43], we investigated the mRNA expression of *TIMP-2* after treatment with MK801 and co-administration of the AChE inhibitor physostigmine in the developing rat brain. Quantitative analysis of *TIMP-2* mRNA expression by real-time PCR (Figure 4) showed a significant down-regulation of *TIMP-2* mRNA expression in the brain of rat pups at 12 (50.9% + 5.2%) and 24 h (31.9% + 3.1%) following MK801 treatment (dark grey bars). A single physostigmine co-application triggered a significant increase of *TIMP-2* expression (light grey bars, 12 h: 139.3% + 15.0%, 24 h: 132.7% + 11.1%).

### 2.2. Discussion

The present study demonstrates that a single co-administration of the AChE inhibitor physostigmine to neonatal rats modulates the expression of the neurotrophin BDNF and leads to the regulation of the extracellular matrix associated molecules MMP-2 and TIMP-2 in the developing brain after pharmacological NMDA receptor blockade. Several groups reported previously that exposure of the rodent brain to NMDA receptor antagonists, including MK801, during a critical period of development causes massive apoptotic neurodegeneration in cortical areas (frontal, retrosplenial, parietal, cingulate cortices), basal ganglia, hippocampus, hypothalamus, and subiculum of the developing brain [1,6,8,56]. Vulnerability to this type of neurotoxicity is maximal during the first postnatal week of the rat. In line with these findings, we previously demonstrated that depletion of neurotrophin-associated signaling is an essential mechanism underlying MK801-induced apoptotic neurodegeneration in the developing rat brain [6,8].

Physostigmine, the pharmacological active component of Calabar beans, is considered to be the prototypical AChE inhibitor [57]. Since we have recently demonstrated a neuroprotective effect of physostigmine in a neonatal rat model of oxygen toxicity [26] we investigated whether this pharmacological intervention might also regulate mechanisms of NMDA receptor-induced neurodegeneration. Physostigmine is used especially for treatment of myasthenia gravis and glaucoma [58], reduces diisopropyl fluorophosphate-induced mortality [59] and improves survival in a model of experimental sepsis [60]. AChE inhibitors enhance the concentration of synaptic acetylcholine, thus improving interaction between neurons of the cholinergic system. Besides this symptomatic mechanism of action there is growing evidence that AChE inhibitors have neuroprotective properties and thereby a damage modifying potential [26,51,61,62]. Administration of AChE inhibitors increases BDNF serum concentrations in Alzheimer’s disease patients reaching control levels and in mice after transient cerebral ischemia and reperfusion [27,28]. BDNF belongs to the family of nerve growth factors and plays an important role in neuronal survival, differentiation, and synaptic plasticity in the central nervous system. MK801 exposure induces a transcriptional down-regulation of *BDNF* mRNA in the neonatal rat brain and leads to a loss of *BDNF* mRNA levels in cultured immature neocortical neurons; whereas BDNF supplementation fully prevented this neurotoxic effect [8]. The impact of physostigmine in this model of pharmacological NMDA receptor blockade indicates that AChE inhibitors have the potential to enhance neurotrophin signaling by an increase of BDNF levels in the developing rat brain. Interestingly, in the presented work (data not shown) and in other studies of oxygen toxicity in the developing brain [26] and adult rats following hypoxia [63], there was no inhibition of AChE activity under control conditions. A possible explanation may be the effect of microRNAs as regulators of cholinergic pathways by targeting neuronal AChE activity under physiological conditions [64–66].

Cortical cell cultures incubated with MMP-2 showed decreased levels of pro-BDNF and increased levels of mature BDNF, suggesting that MMP-2 can mediate the conversion of BDNF [55]. Indeed, the physostigmine-induced increase in BDNF expression coincided with increased enzymatic activity of MMP-2 after non-competitive NMDA receptor blockade. Additionally, in cerebral ischemia, large elevations in MMP-2 activity levels were observed as well [36,41]. Our findings are consistent with other studies detecting an increase of MMP-2 after traumatic brain injury, hypoxia-ischemia and an ischemia reperfusion model [67–69] in the injured brain. In contrast, Uckermann *et al.* did not detect any changes in MMP-2 mRNA or protein expression after treatment with MK801 in the immature brain [70]. These differences can be explained by the higher amount of MK801 administrated in the experimental procedures and the higher protein concentration used in the gelanolytic MMP-2 activity analysis in our study, respectively.

The enzymatic activity of MMP-2 is inactivated by TIMP-2 as pro-MMP-2 forms a complex with TIMP-2, which is cleaved by MMP-14 to activate pro-MMP-2 on the cell surface [43]. In agreement with our results, decreased expressions of TIMP-2 levels were demonstrated after traumatic brain injury in the immature rat brain [69].

Our findings are highly relevant from a clinical point of view because compounds acting as NMDA receptor antagonists are frequently applied as sedatives and anesthetics in neonatal, pediatric and obstetric medicine. Based on our findings, we hypothesize that AChE inhibition may be suitable as a preventative neuroprotective strategy in neonatal medicine to counteract toxicity in cases where administration of NMDA receptor blockers cannot be substituted.

## 3. Experimental Section

### 3.1. Animals and Experimental Procedure

#### 3.1.1. Drug Treatment

Sex-matched Wistar rat pups (Charité-Universitätsmedizin Berlin, Berlin, Germany), six-day-old (P6), weighing 10–16 g, received intraperitoneal (i.p.) injections of the non-competitive NMDA receptor antagonist dizocilpine ((+)MK801, Tocris, Bristol, UK; 0.5 mg/kg 0, 8, and 16 h) or vehicle in combination with physostigmine (100 μg/kg Anticholium^®^, Dr. Franz Köhler Chemie, Alsbach-Hähnlein, Germany) at 0 h. All procedures were approved by the local state authorities for animal welfare and followed institutional guidelines.

#### 3.1.2. Tissue Sampling

Six, twelve or twenty four hours following the last injection of MK801, Wistar rat pups (*n* = 6–8 per group) were anesthetized with an i.p. injection of ketamine (50 mg/kg) and xylazine (10 mg/kg) and were transcardially perfused with normal saline solution. After decapitation the olfactory bulb and cerebellum were removed, brain hemispheres were snap-frozen in liquid nitrogen and stored at −80 °C until further analysis (AChE activity assay, quantitative real-time PCR and gelatin zymography).

### 3.2. AChE Activity Assay

AChE activity was measured using the Amplex^®^ Red Acetylcholine/Acetylcholinesterase Assay kit (Invitrogen, Karlsruhe, Germany). For the measurement of AChE activity, 0.1 mL of protein extract (2 μg proteins per sample in assay buffer) was spotted in duplicate into 96-well microplates. An AChE standard curve was used in each experiment. In each well, 0.1 mL assay buffer (50 mM Tris-HCl, pH 7.5) containing 0.4 mM Amplex Red reagent, 2 U/mL horseradish peroxidase, 0.2 U/mL choline oxidase, and 0.1 mM acetylcholine was added. After incubation, the fluorescence was determined in a fluorescence microplate reader (Infinite^®^ M200, Tecan, Crailsheim, Germany) using 560 nm excitation and 590 nm emission wavelength. The enzymatic activity was determined using the software provided by the manufacturer (Magellan V 6.3, Tecan) and expressed as units per milligram protein.

### 3.3. Semiquantitative Real-Time PCR

Total cellular RNA was isolated from snap-frozen tissue by acidic phenol/chloroform extraction and DNase I treated (Roche Diagnostics, Mannheim, Germany); 2 μg of RNA was reverse transcribed with Moloney murine leukemia virus reverse transcriptase (Promega, Madison, WI, USA) in 25 μL of reaction mixture. The resulting cDNA (1 μL) was amplified by real-time PCR. The PCR product of *BDNF* and *TIMP-2* was quantified in real-time, using a dye-labeled fluorogenic reporter oligonucleotide probe and gene specific primers (Table 1). All probes were labeled at their 5′ ends with the reporter dye 6-carboxy-fluoresceine (FAM), at their 3′ ends with the quencher dye 6-carboxy-tetramethylrhodamine (TAMRA) and were purchased from Metabion (Munich, Germany). *β-actin* was used as internal standard. Real-time PCR and detection were performed in triplicate and repeated three times for each sample using a total reactive volume of 13 μL which contained 6.5 μL of 2× TaqMan Universal PCR Master Mix (Applied Biosystems, Foster City, CA, USA), 2.5 μL of 1.25 μM oligonucleotide mix, 0.5 μL (0.5 μM) of probe (Metabion). The PCR amplification was performed in 96-well optical reaction plates for 40 cycles with each cycle at 94 °C for 15 s and 60 °C for 1 min. Each plate included at least three “no template controls”. The expression of *BDNF*, *TIMP-2* and *β-actin* was analyzed with the real-time PCR ABI Prism 7500 sequence detection system (Applied Biosystems) according to the 2^−ΔΔ^*^C^*^t^ method [26].

### 3.4. Immunoblotting

Snap-frozen tissue was homogenized in RIPA (radioimmunoprecipitation assay) buffer (1% NP40, 0.5% sodium deoxycholate, 0.1% SDS, 1 mM EDTA, 1 mM EGTA, 1 mM Na_3_VO_4_, 20 mM NaF, 0.5 mM DTT, 1 mM PMSF and protease inhibitor cocktail in PBS pH 7.4). The homogenate was centrifuged at 1050× *g* (4 °C) for 10 min, and the microsomal fraction was subsequently centrifuged at 17,000× *g* (4 °C) for 20 min. After collecting the supernatant, protein concentrations were determined using the bicinchoninic acid kit (Interchim, Montluçon, France). Protein extracts (25 μg per sample) were denaturated in Laemmli sample loading buffer at 95 °C, separated by 15% sodium dodecyl sulfate polyacrylamide gel electrophoresis and electrotransferred in transfer buffer to a nitrocellulose membrane (0.2 μm pore, Protran; Schleicher & Schüll, Dassel, Germany). Nonspecific protein binding was prevented by treating the membrane with 5% nonfat dry milk in Tris-buffered saline/0.1% Tween 20 for 2 h at room temperature. Equal loading and transfer of proteins was confirmed by staining the membranes with Ponceau S solution (Fluka, Buchs, Switzerland). The membranes were incubated overnight at 4 °C with rabbit polyclonal anti-BDNF (14 kDa; 1:500; Santa Cruz Biotechnology, Heidelberg, Germany). Secondary incubations were performed with horseradish peroxidase-linked anti-rabbit (1:2000; Amersham Biosciences, Bucks, UK) antibody. Positive signals were visualized using enhanced chemiluminescence (ECL; Amersham Biosciences) and quantified using a ChemiDoc™ XRS+ system and the software Quantity One^®^ (Bio-Rad, Munich, Germany). Membranes were stripped, then washed, blocked and reprobed overnight at 4 °C with mouse anti-β-actin monoclonal antibody (42 kDa; 1:10,000; Sigma-Aldrich, Taufkirchen, Germany).

### 3.5. Gelatin Zymography

Snap-frozen tissue was homogenized in working buffer (150 mM NaCl, 5 mM CaCl_2_, 0.05% Brij 35, 1% Triton X-100, 0.02% NaN_3_, 50 mM Tris-HCl pH 7.4) on ice. Homogenate was centrifuged at 12,000× *g* for 5 min and the protein concentration of the supernatant was measured with a bicinchoninic acid assay (Interchim). 500 μg of protein were used for enzyme enrichment and purification with Gelatin Sepharose™ 4B (GE Healthcare, Uppsala, Sweden) and eluted in 150 μL working buffer containing 10% dimethylsulfoxide. Subsequently samples were loaded on a 7.5% SDS polyacrylamide gel embedded with 0.1% gelatin and electrophoresis was carried out at 120 V for 2 h at 4 °C. Following electrophoresis, the gel was washed twice for 15 min with 2.5% Triton X-100 to remove SDS and to re-nature the gelatinases. For activation of the enzymes, the gel was incubated in substrate buffer (5 mM CaCl_2_, 25 μM ZnCl_2_, 5% Triton X-100, 0.1% NaN_3_, 250 mM Tris-HCl pH 7.4) for 24 h at 37 °C with gentle shaking. The gel was stained with Coomassie Blue R250 in 10% acetic acid and 30% methanol for 2 h and destained briefly in the same solution without dye until white bands representing proteolytic activities were clearly visible. The gel was stabilized in 5% glycerol and 20% methanol for 15 min. Quantification of MMP-2 band density was carried out using a ChemiDoc™ XRS+ system and the software Quantity One^®^ (Bio-Rad).

### 3.6. Statistical Analysis

Data were analyzed using GraphPad Prism 6 (GraphPad Software, La Jolla, CA, USA) and are presented as mean values + standard deviation (SD). Group effects were assessed by One-way analysis of variance (ANOVA) followed by Bonferroni post-hoc test. *p*-values of <0.05 were considered statistically significant.

## 4. Conclusions

The present study suggests that AChE may work as a therapeutic target to limit NMDA receptor antagonist-induced injury in the developing brain. Our results show that a single co-application of the AChE inhibitor physostigmine modulates NMDA receptor blockade-induced changes in the levels of BDNF, MMP-2 and TIMP-2. Our findings indicate that additional non-cholinergic functions of AChE might be responsible for the effects of pharmacologic NMDA receptor antagonists to the developing brain.

## Figures and Tables

**Table 1.** Sequences of oligonucleotides and gene locus.

Gene		Oligonucleotide sequences 5′-3′	
*BDNF*	Forward	TCAGCAGTCAAGTGCCTTTGG	M61175
	reverse	CGCCGAACCCTCATAGACATG	
	probe	CCTCCTCTGCTCTTTCTGCTGGAGGAATACAA	
*TIMP-2*	Forward	GGCAACCCCATCAAGAGGAT	NM_021989
	reverse	GGGCCGTGTAGATAAATTCGAT	
	probe	AGATGTTCAAAGGACCTGAC	
*β-actin*	Forward	GTACAACCTCCTTGCAGCTCCT	NM_031144
	reverse	TTGTCGACGACGACGGC	
	probe	CGCCACCAGTTCGCCATGGAT	
